# Green tobacco sickness among tobacco farmers in southern Brazil

**DOI:** 10.1002/ajim.22307

**Published:** 2014-02-13

**Authors:** Anaclaudia G. Fassa, Neice M.X. Faria, Rodrigo D. Meucci, Nadia Spada Fiori, Vanessa Iribarrem Miranda, Luiz Augusto Facchini

**Affiliations:** ^1^ Social Medicine DepartmentPostgraduate Program in Epidemiology, Federal University of PelotasBrazil; ^2^ Municipal Health Department of Bento GonçalvesRio Grande do SulBrazil

**Keywords:** green tobacco sickness, tobacco farming, poisoning, epidemiology, prevalence

## Abstract

**Background:**

Despite being the second largest tobacco producer in the world, Brazil does not have prevalence studies about green tobacco sickness (GTS).

**Methods:**

A cross‐sectional study was carried out on a sample of Brazilian tobacco workers. The sample was described according to socio‐demographic, behavioral, and occupational variables. Gender‐stratified multivariate analyses examined variables associated with GTS.

**Results:**

GTS prevalence among men in the previous month was 6.6%, while among women it was 11.9%. Among men, age, being a non‐smoker, hanging tobacco sticks in the barn, harvesting wet leaves, and exposure to physical exertion were risk factors for GTS. Among women, tying hands of tobacco, transporting bales, harvesting wet leaves, having had contact with pesticides, and exposure to physical exertion were positively associated with GTS.

**Conclusion:**

Research is required to improve methods for GTS screening, as well as the ability to distinguish GTS from pesticide poisoning. Health professionals should be trained to diagnose and treat GTS. Am. J. Ind. Med. 57:726–735, 2014. © 2014 The Authors. American Journal of Industrial Medicine Published by Wiley Periodicals, Inc.

## INTRODUCTION

Tobacco is cultivated in more than 100 countries, however, six countries account for 2/3 of all production: China, Brazil, India, USA, Malawi, and Indonesia [Schmitt et al., 2007]. It is estimated that more than 30 million farmworkers are involved in tobacco production around the world [Schmitt et al., 2007]. Brazil is the second leading tobacco producing country worldwide, and in southern Brazil more than 220,000 families are directly involved in tobacco cultivation [IBGE, 2012].

Green tobacco sickness (GTS) is acute nicotine poisoning caused by transdermal absorption of nicotine in tobacco farmers and farmworkers as they come into contact with green tobacco leaves. This illness is characterized by the occurrence of dizziness or headache and nausea or vomiting [Schmitt et al., 2007; Arcury et al., 2008], although some studies have presented other criteria for defining GTS [Onuki et al., 2003; Parikh et al., 2005].

GTS has been described in tobacco farmworkers in several regions in the USA, Japan, India, Italy, and more recently in Brazil, where two case‐control studies were carried out [Quandt et al., 2000; Arcury et al., 2001a; Parikh et al., 2005; Oliveira et al., 2010; Bartholomay et al., 2012]. The few existing studies show great variability in GTS prevalence of between 8.2% and 47% during the tobacco‐growing season [Quandt et al., 2000; Arcury et al., 2001a; Parikh et al., 2005]. One study presented GTS incidence density of 1.88 days per 100 days [Arcury et al., 2001a]. Another study found an incidence rate of 10 cases of hospital‐treated GTS per 1,000 workers in 2 months [CDC, 1993]. High prevalence variability may be related to methodological differences in the studies or differences in the work process. Activities associated with GTS are pruning, harvesting, curing, and baling [Arcury et al., 2001a; Oliveira et al., 2010]. Being a non‐smoker, lacking experience in tobacco work, not using protective clothes, having skin cuts or rashes, and working in wet clothes and shoes are also factors associated with GTS [Quandt et al., 2000; Arcury et al., 2008; Oliveira et al., 2010].

Despite the growing economical importance of tobacco cultivation in developing countries and the great number of workers involved in this activity, few studies have evaluated GTS prevalence. Furthermore, many of the existing studies have a small sample size and low statistical power to analyze associated factors. In Brazil there are no data on GTS prevalence. Therefore the objective of this study is to identify GTS prevalence and its associated factors in São Lourenço do Sul. This city is located in Rio Grande do Sul (RS) State, which accounts for more than 50% of Brazilian tobacco production. This study has relevance in places producing mainly Virginia tobacco through manual farming by families, as is the case of southern Brazil.

## METHODS

A cross‐sectional study was carried out among tobacco workers in São Lourenço do Sul (SLS)––RS during the harvest season, January–March 2011.

The sample size took into account the following parameters: (a) estimation of prevalence: 95% confidence interval, 12% estimated prevalence of GTS, and acceptable error of 1.5 pp; (b) evaluation of associated factors: 80% statistical power, 10:1 ratio between exposed and non‐exposed people regarding the use of personal protective equipment and relative risk of 1.8. A further 10% was added to account for missing data and refusals, and 15% for confounding factors. The final estimated sample size was 2,584 workers.

All tobacco farms issue invoices for tobacco sales. Most tobacco farmers issue at least one invoice a year for tobacco sales to guarantee their retirement rights, and because of this more than one worker often issue invoices at each farm. Of the total of 3,852 invoices issued in SLS in the 2009 season, 1,100 invoices were randomly sampled from different farms and all workers living in the selected farms were interviewed.

Invoices were considered to be ineligible if they related to individuals who were not tobacco workers, lived on a farm with no tobacco production, or those who lived in urban areas, or who had moved to other cities. Invoices raised by subjects who had been tobacco workers in 2009 but had later quit were excluded and replaced by invoices from the next nearest tobacco farm. Although the invoices refer to individual tobacco farmers, the unit of selection was the farm. The use of invoices assured that the sample of SLS tobacco growers was representative. The community health workers involved in this study located the selected farms.

### Data Collection

At each farm all subjects that had been working in tobacco production for at least 15 hr a week were interviewed. Two types of questionnaire were used: one for the farm and another for individuals.

The farm questionnaire included socio‐economic variables such as the amount of tobacco produced in the previous year, and the extent of agricultural diversification (milk production, honey, and other crops).

The individual questionnaire collected demographic (gender, age), socio‐economic (educational background), behavioral (smoking and alcohol abuse), and occupational information. Individuals who reported smoking one or more cigarette a day for more than 1 month were considered smokers, while those who had quit smoking for more than a month were considered ex‐smokers. Alcohol abuse was evaluated through the CAGE test, which included four questions [Ewing, 1984]. Those who answered “yes” to two or more questions were considered positive.

Variables relating to work experience in tobacco production and hours of work during the tobacco‐growing season were collected. We investigated activities such as topping (removing tobacco flower), wet leaf harvesting, holding leaves under the arms, supplying tobacco curing barns with loose leaves, climbing high into the barn, hanging tobacco sticks in the barn, controlling barn temperature and humidity, tying hands of tobacco, baling, and transportation of bales.

The hazards evaluated were contact with pesticides in the last year, activities requiring considerable physical exertion, contact with tobacco dust, and entering the barn during the curing process. Using protective gloves and clothing, as well as working in wet clothes during tobacco harvesting were also investigated.

Green tobacco sickness was characterized by the occurrence of dizziness or headache and nausea or vomiting within 2 days after tobacco harvesting [Schmitt et al., 2007; Arcury et al., 2008].

The questionnaires were administered by trained interviewers using personal digital assistants (PDA). A short version of the questionnaire was re‐administered to 10% of the interviewees for the purpose of quality control.

### Analysis

Smoking is a variable negatively associated with GTS. However, prevalence of smoking among women was low, hindering its inclusion in the multivariate analysis. The study provided statistical power to evaluate the impact of smoking on GTS among men and gender‐stratified analyses were therefore carried out. Subjects aged 18 or older were included.

Data analysis included a description of the studied sample according to independent variables using the chi‐squared test to evaluate heterogeneity between men and women. GTS prevalence was calculated according to different time frames: in the previous year, previous month, and previous week.

Crude and adjusted analyses of the association between independent variables and GTS in the previous month were carried out using Poisson regression with backward selection taking into account the design effect of 1.29. Wald test of heterogeneity or linear trend was used. Multivariable analysis followed a hierarchical model [Victora et al., 1997], which included demographic and socio‐economic variables on the first level; occupational (work hours and type of activities performed in the previous year), and behavioral variables on the second level; workloads on the third level; and wearing protective clothing during harvest on the fourth level. Variables with *P*‐value ≤0.2 were maintained in the models and those with <0.05 were considered to be associated.

The Research Ethics Committee of the Federal University of Pelotas approved this study and all the interviewees signed an informed consent. Subjects with symptoms of GTS were referred to public health services.

## RESULTS

2,469 individuals (1,464 males and 1,005 females) on the 912 farms were included in the study. 5.9% of the subjects were lost or refused to participate.

Some 44% of workers had produced 5–10 tons of tobacco in the previous year. Approximately 25% had produced milk, 18% had produced honey, and 27% had cultivated at least one crop besides tobacco (Table I).

**Table I:** Demographic and Socio‐Economic Description of the Tobacco Farmer Sample Stratified by Gender. Brazil, 2013

Variable	Male		Female		P*
	N	% (95% CI)	N	% (95% CI)	
Age (years)					0.08
18–29	403	27.5 (25.2–29.8)	294	29.3 (26.4–32.1)	
30–39	342	23.4 (21.2–25.5)	229	22.8 (20.2–25.4)	
40–49	316	21.6 (19.5–23.7)	246	24.5 (21.9–27.1)	
≥50	403	27.5 (25.2–29.8)	236	23.5 (20.9–26.1)	
Schooling					0.02
0–4	645	44.1 (41.5–46.6)	441	43.9 (40.8–47.0)	
5–8	732	50.0 (47.4–52.6)	473	47.1 (44.0–50.2)	
≥9	87	5.9 (4.7–7.2)	91	9.1 (7.3–10.8)	
Amount of tobacco produced (kg)					0.2
1–2,500	90	6.2 (4.9–7.4)	70	7.0 (5.4–8.6)	
2,501–5,000	396	27.2 (24.9–29.5)	290	29.1 (26.2–31.9)	
5,001–10,000	638	43.8 (41.3–46.4)	438	43.9 (40.8–47.0)	
10,001–36,000	331	22.7 (20.6–24.9)	200	20.0 (17.6–22.5)	
Expenses with road tax––R$					0.7
Free	151	10.6 (9.0–12.2)	113	11.5 (9.5–13.5)	
≤500	558	39.1 (36.6–41.7)	397	40.6 (37.5–43.6)	
501–1,000	495	34.7 (32.2–37.2)	319	32.6 (29.6–35.5)	
≥1,001	222	15.6 (13.7–17.4)	150	15.3 (13.1–17.6)	
Milk production					0.3
No	1,080	74.2 (71.9–76.4)	721	72.2 (69.4–75.0)	
Yes	376	25.8 (23.6–28.1)	278	27.8 (25.0–30.6)	
Honey production					0.7
No	1,187	81.5 (79.5–83.5)	820	82.1 (79.7–84.5)	
Yes	269	18.5 (16.5–20.5)	179	17.9 (15.5–20.3)	
Other crops production					0.4
No	689	47.5 (45.0–50.1)	487	48.9 (45.8–52.1)	
One crop	396	27.3 (25.0–29.6)	267	26.8 (24.1–29.6)	
Two crops	239	16.5 (14.6–18.4)	172	17.3 (14.9–19.6)	
Three or more crops	125	8.7 (7.2–10.1)	69	7.0 (5.3–8.5)	
Livestock production (kind)					0.8
No	1,060	72.9 (70.7–75.2)	734	73.6 (70.9–76.4)	
Up to one	258	17.8 (15.8–19.7)	178	17.9 (15.5–20.2)	
Two or more	135	9.3 (7.8–10.8)	85	8.5 (6.8–10.3)	

95% CI: confidence interval.

* Chi‐squared test of heterogeneity.

There was a similar proportion of men in the 18–29 and ≥50 age groups (27.5%, respectively), whereas most of the women were aged between 18 and 29 (29.3%). Regarding education, 50.0% of men, and 47.1% of women had attended school for 5–8 years (Table I). More than 30% of men were smokers while among women this proportion was 3.2%. The CAGE test was positive for 4.7% of males and 0.1% of females (Table II).

**Table II:** Behavioral and Occupational Description of the Tobacco Farmer Sample Stratified by Gender. Brazil, 2013

	Male		Female		P*
	N	% (95% CI)	N	% (95% CI)	
Smoking					<0.001
No	1,007	68.8 (66.4–71.2)	974	96.9 (95.8–98.0)	
1–9 cigarettes/day	110	7.5 (6.2–8.9)	17	1.7 (0.9–2.5)	
≥10 cigarettes/day	347	23.7 (21.5–25.9)	14	1.4 (0.7–2.1)	
CAGE test					<0.001
No	1,395	95.3 (94.2–96.4)	1,004	99.9 (99.7–100.0)	
Yes	69	4.7 (3.6–5.8)	1	0.1 (0.0–0.2)	
Time working with tobacco (years)					0.3
<5	86	5.9 (4.7–7.1)	61	6.1 (4.6–7.6)	
5–9	371	25.4 (23.1–27.6)	250	24.9 (22.2–27.6)	
10–14	244	16.7 (14.8–18.6)	196	19.5 (17.1–22.0)	
15–19	211	14.4 (12.6–16.2)	151	15.0 (12.8–17.3)	
20–24	217	14.8 (13.0–16.7)	147	14.7 (12.5–16.8)	
≥25	334	22.8 (20.7–25.0)	198	19.8 (17.3–22.2)	
Working hours during tobacco‐growing season					<0.001
≤8 hr	124	8.5 (7.1–9.9)	195	19.5 (17.0–21.9)	
9–12 hr	805	55.2 (52.6–57.7)	556	55.4 (52.3–58.5)	
13–1 hr	530	36.3 (33.9–38.8)	252	25.1 (22.4–27.8	
Supplying tobacco curing barns with loose leaves					0.3
No	1240	84.8 (82.9–86.6)	866	86.2 (84.0–88.3)	
Sometimes/always	223	15.2 (13.4–17.1)	139	13.8 (11.7–16.0)	
Climbing high into the curing barn					<0.001
No	383	26.2 (23.9–28.4)	842	83.8 (81.5–86.1)	
Sometimes	99	6.8 (5.5–8.1)	70	7.0 (5.4–8.5)	
Always	981	67.1 (64.6–69.5)	93	9.3 (7.5–11.0)	
Hanging tobacco sticks in the curing barn					<0.001
No	528	36.1 (33.6–38.6)	179	17.8 (15.4–20.2)	
Sometimes	415	28.4 (26.0–30.7)	91	9.1 (7.3–10.8)	
Always	520	35.5 (33.1–38.0)	735	73.1 (70.4–75.9)	
Controlling curing barn temperature					<0.001
No	124	8.5 (7.1–9.9)	291	29.0 (26.1–31.8)	
One period	376	25.7 (23.5–28.0)	533	53.0 (49.9–56.1)	
Two periods	961	65.8 (63.3–68.2)	181	18.0 (15.6–20.4)	
Tobacco classification					<0.001
No	198	13.5 (11.8–15.3)	87	8.7 (6.9–10.4)	
Sometimes	94	6.4 (5.2–7.7)	49	4.9 (3.5–6.2)	
Always	1,171	80.0 (78.0–82.1)	869	86.5 (84.3–88.6)	
Topping					0.01
No	123	8.4 (7.0–9.8)	121	12.0 (10.0–14.0)	
Sometimes	88	6.0 (4.8–7.2)	70	7.0 (5.4–8.5)	
Always	1,253	85.6 (83.8–87.4)	814	81.0 (78.6–83.4)	
Wet leaves harvest					0.8
No	414	28.3 (26.0–30.6)	279	27.8 (25.1–30.6)	
Yes	1,048	71.7 (69.4–74.0)	723	72.2 (69.4–74.9)	
Bottom leaves harvest					0.01
No/sometimes	65	4.4 (3.4–5.5)	71	7.1 (5.5–8.6)	
Always	1,399	95.6 (94.5–96.6)	934	92.9 (91.3–94.5)	
Top leaves harvest					0.02
No	61	4.2 (3.1–5.2)	62	6.2 (4.7–7.7)	
Yes	1,400	95.8 (94.8–96.9)	942	93.8 (92.3–95.3)	
Holding leaves under the arms					<0.001
No	64	4.4 (3.3–5.4)	370	36.9 (33.8–39.8)	
Sometimes	45	3.1 (2.2–4.0)	116	11.5 (9.6–13.5)	
Always	1,354	92.5 (91.2–93.9)	519	51.6 (48.5–54.7)	
Tying hands of tobacco					0.5
No/sometimes	210	14.3 (12.6–16.2)	135	13.4 (11.3–15.5)	
Always	1,253	85.6 (83.8–87.4)	870	86.6 (84.4–88.7)	
Baling tobacco					<0.001
No	70	4.8 (3.7–5.9)	155	15.5 (13.2–17.7)	
Sometimes	117	8.0 (6.6–9.4)	140	14.0 (11.8–16.1)	
Always	1,275	87.2 (85.5–88.9)	707	70.5 (67.7–73.4)	
Baling transportation					<0.001
No	702	48.0 (45.4–50.6)	850	84.6 (82.3–86.8)	
Sometimes	234	16.0 (14.1–17.9)	70	7.0 (5.4–8.5)	
Always	526	36.0 (33.5–38.4)	85	8.5 (6.7–10.2)	
Tasks that require a lot of physical exertion					<0.001
No	360	24.6 (22.4–26.8)	532	53.0 (50.0–56.1)	
Yes	1,104	75.4 (73.2–77.6)	472	47.0 (43.9–50.1)	
Entering the hot barn					<0.001
No	661	45.2 (42.6–47.7)	795	79.2 (76.7–81.7)	
Yes	803	54.8 (52.3–57.4)	209	20.8 (18.3–23.3)	
Contact with tobacco dust					<0.001
No	90	6.1 (4.9–7.4)	69	6.9 (5.3–8.4)	
Little	635	43.4 (40.8–45.9)	355	35.4 (32.4–38.3)	
Too much	739	50.5 (47.9–53.0)	580	57.8 (54.7–60.8)	
Working in wet clothes					0.001
No	168	11.5 (9.9–13.1)	162	16.2 (13.9–18.5)	
Yes	1,293	88.5 (86.9–90.1)	838	83.8 (81.5–86.1)	
Contact with pesticides					<0.001
No	237	16.2 (14.3–18.1)	594	59.2 (56.1–62.2)	
Yes	1,226	83.8 (81.9–85.7)	410	40.8 (37.8–43.9)	
Contact with kerosene/thinner					0.001
No	1,385	94.6 (93.4–95.8)	978	97.4 (96.4–98.4)	
Yes	79	5.4 (4.2–6.5)	26	2.6 (1.6–3.6)	
Contact with paint					0.8
No	1,387	94.7 (93.6–95.9)	949	94.5 (93.1–95.9)	
Yes	77	5.3 (4.1–6.4)	55	5.5 (4.1–6.9)	
Contact with gasoline					<0.001
No	267	18.2 (16.3–20.2)	907	90.2 (88.4–92.1)	
Yes	1,197	81.8 (79.8–83.7)	97	9.7 (7.8–11.5)	
Contact with disinfectant					<0.001
No	1,307	89.3 (87.7–90.9)	313	31.1 (28.3–34.0)	
Yes	157	10.7 (9.1–12.3)	691	68.8 (65.9–71.6)	
Use of protective clothes					<0.001
No	712	48.7 (46.2–51.3)	406	40.5 (37.5–43.6)	
Sometimes	403	27.6 (25.3–29.9)	242	24.2 (21.5–26.8)	
Always	346	23.4 (21.5–25.9)	353	35.3 (32.3–38.2)	
Use of protective gloves					<0.001
No	974	66.7 (64.2–69.1)	406	40.6 (37.6–43.7)	
Sometimes	124	8.5 (7.1–9.9)	97	9.7 (7.9–11.5)	
Always	363	24.9 (22.6–27.1)	497	49.7 (46.6–52.8)	

95% CI, confidence interval.

* Chi‐squared test of heterogeneity.

Around 90% of males and 80% of females worked more than 8 hr per day. Predominantly male activities included: topping (85.6%), climbing high into the barn (67.1%), controlling barn temperature and humidity (65.8%), holding leaves under the arms (92.5%), baling (87.2%), and transporting bales (36.0%). Predominantly female activities were: hanging tobacco sticks (73.1%), classifying tobacco (86.5%), and topping (81.0%) (Table II).

There was no gender difference in exposure to wet tobacco leaves. However, 35.3% of the women reported always using protective clothing during harvest, while for men this proportion was 23.4%. There was 49.7% protective glove use among women and 24.9% among men. More than 80% of men and women reported working in wet clothes during harvest (Table II).

83.8% of men and 40.8% of women reported exposure to pesticides in the previous year. Males experienced higher exposure to activities requiring physical exertion and higher exposure to entering the hot barn than females did (Table II).

GTS prevalence among men in the previous year, month, and week was 9.6%, 6.6%, and 3.3%, respectively, while among women it was 15.7%, 11.9%, and 6.6%.

As seen in Table III, GTS prevalence in the previous month among males aged 30–39 was 9.1%, while it was 4.0% among men aged ≥50. GTS prevalence among men who harvested wet tobacco leaves was 7.9% while prevalence among those who did not harvest them was 3.1% (Table III).

**Table III:** Green Tobacco Sickness (GTS): Prevalence and Associated Factors Among Men. Brazil, 2013

Variable	GTS %	Crude			Adjusted		
		PR	(95% CI)	*P*	PR	(95% CI)	*P*
1st level							
Age (years)				0.03**			0.03**
18–29	6.9	1.75	0.96–3.16		1.78	0.98–3.22	
30–39	9.1	2.29	1.28–4.09		2.19	1.22–3.93	
40–49	6.7	1.68	0.91–3.12		1.63	0.89–3.00	
≥50	4.0	1	––		1	––	
Amount of tobacco produced (kg) (farm)				0.18**			0.07**
1–2,500	7.7	1			1		
2,501–5,000	8.6	1.12	0.50–2.48		1.06	0.48–2.33	
5,001–10,000	5.5	0.71	0.33–1.55		0.61	0.29–1.31	
10,001–36,000	6.1	0.79	0.34–1.85		0.68	0.29–1.55	
Milk production (farm)				0.01*			0.005*
No	7.7	1	––		1	––	
Yes	2.9	0.45	0.25–0.78		0.45	0.26–0.78	
Honey production (farm)				0.03*			0.06*
No	5.9	1	––		1	––	
Yes	9.7	1.34	1.02–1.77		1.32	0.99–1.74	
Other crops production (farm)				0.15**			0.04*
No	6.0	1	––		1	––	
One crop	5.3	0.89	0.51–1.56		0.94	0.53–1.64	
Two crops	10.5	1.76	1.08–2.87		1.83	1.15–2.93	
Three or more crops	6.4	1.07	0.52–2.24		1.01	0.48–2.14	
2nd level							
Smoking				0.04**			0.04*
No	7.3	1.95	1.06–3.59		2.10	1.12–3.95	
1–9 cigarettes/day	8.2	2.17	1.00–4.70		2.47	1.10–5.53	
≥10 cigarettes/day	3.8	1	––		1	––	
Hanging tobacco sticks				0.05**			0.02**
No	5.7	1	––		1	––	
Sometimes	4.8	0.84	0.48–1.48		0.96	0.55–1.68	
Always	8.8	1.55	1.00–2.39		1.71	1.10–2.66	
Wet leaves harvest				0.001*			0.001*
No	3.1	1	––		1	––	
Yes	7.9	2.52	1.44–4.43		2.63	1.50–4.63	
3rd level							
Working in wet clothes				0.1*			0.15*
No	3.6	1	––		1	––	
Yes	7.0	1.95	0.89–4.29		1.80	0.79–4.09	
Tasks that require a lot of physical exertion				0.001*			0.01*
No	2.8	1	––		1	––	
Yes	8.1	2.80	1.46–5.37		2.33	1.18–4.61	
Contact with tobacco dust				0.003**			0.05**
No	2.2	1	––		1	––	
Little	5.0	2.27	0.55–9.31		1.94	0.47–7.95	
Too much	8.4	3.78	0.94–15.1		2.71	0.65–11.2	

PR, prevalence rate.

95% CI, confidence interval.

* Wald test of heterogeneity.

** Wald test of linear trend.

In the adjusted analysis age was inversely associated with GTS among men. Those aged 30–39 presented the highest risk (PR 2.19). Milk production showed protection against GTS (PR 0.45), and those who were growing two crops apart from tobacco showed higher risk than those producing no other crops (PR 1.83).

Non‐smokers and individuals who smoked 1–9 cigarettes a day showed higher risk of GTS compared to those who smoked 10 or more cigarettes a day (Table III).

Repeatedly hanging tobacco sticks in the barn (PR 1.71), harvesting wet tobacco leaves (PR 2.63), and exposure to activities requiring considerable physical exertion (PR 2.33) were positively associated with GTS among men (Table III).

More than 14% of women who harvested wet tobacco leaves reported symptoms of GTS, whereas prevalence was 5.8% among those not exposed to wet leaves. GTS prevalence was 50% higher in women exposed to physical exertion than in unexposed women (Table IV).

**Table IV:** Green Tobacco Sickness (GTS): Prevalence and Associated Factors Among Women. Brazil, 2013

Variable	GTS %	Crude			Adjusted		
		PR	(95% CI)	*P*	PR	(95% CI)	*P*
1st level							
Milk production (farm)				0.13*			0.12*
No	13.0	1	––		1	––	
Yes	9.4	0.73	0.48–1.09		0.73	0.48–1.09	
2nd level							
Hours of work during tobacco‐growing season				0.02**			0.07**
Up 8 hr	8.3	1	––		1	––	
9–12 hr	11.5	1.38	0.82–2.32		1.18	0.70–1.97	
13–18 hr	15.5	1.86	1.06–3.26		1.61	0.93–2.80	
Tying hands of tobacco				0.03*			0.04*
No/sometimes	6.0	1	––		1	––	
Always	12.8	2.15	1.08–4.28		2.00	1.01–3.97	
Bales transportation				0.01**			0.02**
No	10.9	1	––		1	––	
Sometimes	12.9	1.18	0.63–2.20		1.14	0.61–2.13	
Always	20.9	1.92	1.21–3.05		1.86	1.18–2.94	
Wet leaves harvest				<0.001*			0.001*
No	5.8	1	––		1	––	
Yes	14.3	2.46	1.50–4.04		2.31	1.42–3.75	
3rd level							
Working in wet clothes				0.01*			0.08*
No	5.0	1	––		1	––	
Yes	13.3	2.67	1.33–5.34		1.82	0.93–3.57	
Contact with pesticides last year				0.04*			0.02*
No	8.8	1	––		1	––	
Yes	16.4	2.13	1.04–4.37		1.48	1.05–2.09	
Tasks that require a lot of physical exertion				<0.001*			0.02*
No	8.1	1	––		1	––	
Yes	16.1	1.98	1.40–2.81		1.56	1.09–2.23	
4th level							
Use of protective clothes				0.08**			0.15**
No	13.6	1	––		1	––	
Sometimes	12.8	0.94	0.62–1.43		0.88	0.58–1.31	
Always	9.4	0.69	0.45–1.04		0.73	0.49–1.11	

PR, prevalence rate.

95% CI, confidence interval.

* Wald test of heterogeneity.

** Wald test of linear trend.

In the adjusted analysis for women, repeatedly tying hands of tobacco (PR 2.00), transporting bales (PR 1.86), harvesting wet tobacco leaves (PR 2.31), being in contact with pesticides in the previous year (PR 1.48), and exposure to activities requiring considerable physical exertion (PR 1.56) were positively associated with GTS (Table IV).

Frequent use of protective clothes during harvest was not associated with GTS in both males and females.

## DISCUSSION

GTS prevalence in the previous month among men was 6.6% and 11.9% among women. This prevalence indicates an important impact of GTS on tobacco workers' health during the harvest season, since GTS symptoms are acute and intense, and result in having to take time off work. Furthermore, long‐term effects of nicotine poisoning are not clearly understood [Parikh et al., 2005].

Age was inversely associated with GTS among men. Being a non‐smoker or smoking up to nine cigarettes a day, hanging tobacco sticks in the barn, harvesting wet leaves, and exposure to physical exertion were risk factors for GTS, while producing milk was a protection factor. Among women, tying hands of tobacco, transporting bales, harvesting wet leaves, having had contact with pesticides in the previous year, and exposure to physical exertion were positively associated with GTS.

Higher GTS prevalence among women may be related to biological gender differences. Females have a relatively larger dermal area through which nicotine can be absorbed in relation to their body volume when compared to males [Quandt et al., 2000; Arcury et al., 2003; Widmaier et al., 2003]. Women may also report symptoms more accurately than men. Moreover, gender division of labor in tobacco cultivation, with differences in intensity and frequency of many tasks performed, can play a role in women being at greater risk of GTS.

The inverse association of GTS with men's age is consistent with the literature. The reasons are uncertain but could be related to young workers higher work intensity and their greater involvement with tasks associated with nicotine poisoning. Furthermore, individuals susceptible to nicotine poisoning could avoid tasks with higher exposure or leave tobacco work entirely (healthy worker effect), and some workers may develop tolerance to nicotine [Arcury et al., 2001a]. On the other hand, no association between age and GTS was found among women, reinforcing gender difference in physiological mechanisms of adaptation to nicotine [Arcury et al., 2001a].

The protective effect of milk production among men may indicate that this activity competes with workers' involvement in tobacco production, thus decreasing their exposure. On the other hand, the higher risk found in men who cultivated two crops other than tobacco for trade may indicate an overlap of GTS, and pesticide poisoning symptoms.

The higher risk of GTS for non‐smokers is consistent with the literature and may relate to the development of nicotine tolerance in smokers [Quandt et al., 2000; Arcury et al., 2001a; Schmitt et al., 2007]. Smoking prevalence among women is very low. The lack of association between smoking and GTS in this gender is therefore probably due to low statistical power. Smoking is associated with a variety of chronic diseases and certainly should not be used as a strategy to prevent GTS [Quandt et al., 2000; Arcury et al., 2001a; Schmitt et al., 2007].

Hanging tobacco sticks in the barn was a GTS risk factor for men. This task requires considerable physical exertion and may increase the transdermal absorption of nicotine due to the contact of the green tobacco leaves with sweaty skin. The barn also concentrates tobacco dust, but it is uncertain whether dust inhalation during the curing process inside the barn is related to GTS.

The association between GTS and pesticide use in the previous year raises strong concern about the ability to distinguish between pesticide poisoning and GTS. Potential interactions between nicotine and other chemical compounds were not studied. Alternatively these activities could be a marker of high work intensity and activity diversity. The same explanation could apply for carrying bales of tobacco, which is mainly performed by men but showed risk for GTS among women.

Harvesting wet tobacco is a consistent risk factor for GTS confirmed in this study for both genders. Nicotine is a soluble molecule, thus the water from tobacco plants increases transdermal absorption of nicotine [Schmitt et al., 2007].

The association between exposure to activities that require considerable physical exertion and GTS among men and women may be due to an increase in sweating, heart rate, and vasodilatation resulting from this workload which can increase transdermal nicotine absorption [Quandt et al., 2000].

In disagreement with the literature, the use of protective clothing did not show protective effect for GTS in either gender [Quandt et al., 2000; Arcury et al., 2001a]. In Brazil, quality control of protective clothing is poor and after having been washed only a few times they lose their effectiveness in protecting workers from pesticides [Francischini, 2009]. This situation may be similar regarding protection against nicotine during harvesting. In addition, the question asked about use of protective clothes referred not only to clothing certified as protective protection equipment, but also included clothes made of thick fabric, overalls or plastic. It is probable that many workers use normal clothes, not treated with water‐repellent, as protective clothing.

Only a small number of individuals did not perform pruning or change out of their wet clothes during harvest. As such, the lack of association with GTS, which disagrees with the literature, could be related to low statistical power.

This is one of the few international studies and the only one in Brazil evaluating prevalence of GTS and associated factors in a population‐based sample. Defining GTS by means of a symptom questionnaire has not been validated. The symptoms used to characterize GTS (dizziness or headache and nausea or vomiting within 2 days after tobacco harvesting) [Quandt et al., 2000; Thundiyil et al., 2008], have overlap with heat illness [Fleischer et al., 2013], and great similarity with pesticide poisoning. This may have generated a misclassification of the outcome. Additional research is needed to define GTS more precisely [Parikh et al., 2005].

The symptoms of GTS are common, therefore health care providers should be trained to diagnose and treat the problem. Stratified analyses show important differences in factors associated with GTS among men and women that must be considered when proposing preventive measures.

The harvesting of wet leaves is a consistently strong risk factor for GTS. Although protective clothes might not be a definitive solution to prevent this outcome, they should be systematically evaluated for effectiveness in preventing GTS. Mechanization of harvesting will not eliminate exposure to nicotine but could reduce it. Moreover, the high risks of some activities undertaken in the barn need to be better understood. Studies to evaluate good alternatives for reducing GTS are necessary.
